# Deformation Behavior of AZ31 Magnesium Alloy with Pre-Twins under Biaxial Tension

**DOI:** 10.3390/ma17133377

**Published:** 2024-07-08

**Authors:** Hanshu Dai, Mengmeng Sun, Yao Cheng

**Affiliations:** 1 Key Laboratory for Light-Weight Materials, Nanjing Tech University, Nanjing 210009, China; 2 School of Mechanical Engineering, Nanjing Vocational University of Industry Technology, Nanjing 210023, China; 3 School of Materials Science and Engineering, Chongqing University, Chongqing 400044, China

**Keywords:** AZ31 magnesium alloy, twinning, detwinning, biaxial tension

## Abstract

In the present study, the mechanical response and deformation behavior of a Mg AZ31 plate with different types of pre-twins was systematically investigated under biaxial tension along the normal direction (ND) and transverse direction (TD) with different stress ratios. The results show that significant hardening was observed under biaxial tension. The yield values in the direction of larger stress values were higher than those under uniaxial loading conditions, and the solute atom segregation at twin boundaries generates more obvious strengthening effect. Noting that, for TRH (with cross compression along the rolling direction (RD) and TD and annealing at 180 °C for about 0.5 h) sample, the strength effect of the RD yield stress $\sigma_{\mathrm{RD}}:\sigma_{\mathrm{ND}}$ = 2:1 was higher than that of the ND yield stress under stress ratio $\sigma_{\mathrm{RD}}:\sigma_{\mathrm{ND}}$ = 1:2. There is a complex competition between twinning and detwinning under biaxal tension along the ND and TD of the pre-twinned samples with the variation in the stress ratio along the TD and RD. The variation in the twin volume fractions for all samples under biaxial firstly decreases and then increases with a higher stress ratio along the ND. As for the TDH sample (precompression along the TD and annealing), the changes of the twin volume fraction were lower than that of the TR sample (cross compression along the TD and RD). However, the amplitude of variation in twin volume fraction of the TRH sample is higher than that of the TR sample. This is because the relative activity of detwinning decreases and that of twinning increases, as the ND stress mainly leads to the growth of pre-twins and the TD stress often promotes detwinning of primary twins. With a higher stress ratio along the ND, the activity of twinning deformation increases and that of detwinning decreases.

## 1. Introduction

Magnesium (Mg) alloys have attracted a great deal of research interest because of their potential applications for lightweight structural components [1,2,3]. The main hurdle to the application of Mg alloys is related to their limited room temperature formability. Enhancing the formability of Mg alloys requires a better understanding of the deformation mechanisms and their influence on the overall mechanical behavior. The fundamental deformation modes of hexagonal close packed (HCP) magnesium are slip and twinning [4,5]. The main slip modes of magnesium are basal slip, prismatic slip, and pyramidal slip. Due to the low CRSS, {10–12} extension twins are easy to be activated [6,7] when stretching along the direction parallel to the c-axis of the crystal or compressing along the direction perpendicular to the c-axis of the crystal. In general, the {10–12} twins generated by pre-loading can also be de-twinning during reloading along some specific directions. For example, the compression along the transverse direction (TD) or tension along the normal direction (ND) of a hot-rolled Mg AZ31 plate will be a {10–12} twinning predominant deformation. The activation of {10–12} twins can refine grains and reorient crystallographic orientation, which have been employed to strengthen Mg alloys or tailor the texture of wrought Mg alloy products [2,8]

Deformation behavior of Mg alloys containing twins has been extensively studied. Twinning, re-twinning and the growth of pre-twins can be activated when reloading on the Mg alloys with pre-twinned {10–12} twins [9]. For example, recompression along the rolling direction (RD) of a Mg AZ31 plate with pre-compression along the TD will activate {10–12} twinning in both the matrix and {10–12} twins [10]. When loading along the specific direction of Mg alloys containing {10–12} extension twins, detwinning can also take place [11]. For example, the detwinning of {10–12} twins can be activated under tension along the TD of hot rolled AZ31 plate with pre-compression along the TD. In addition, the detwinning can also take place when compressing along the ND of hot-rolled AZ31 plate with pretension along the ND. This is because the pretension along the ND or precompression along the TD of AZ31 plate will activate a large number of {10–12} twins, and a reverse reloading often leads to detwinning of these {10–12} twins [11]. As detwinning does not need nucleation, the activation stress for detwinning is considered to be much lower than that for twinning nucleation [12]. The low activation stress of detwinning often results in quite a low yield stress in a detwinning predominant deformation. Similar to twinning, detwinning also reorients the crystallographic orientation of the twinned region by about 86.3° [13].

Previously, research on the deformation behavior of Mg alloys containing pre-twinned {10–12} twins was mainly focused on uniaxial tension or compression. Generally, AZ31 alloy is a typical wrought magnesium alloy that is used as a rolling and extrusion product. Therefore, the plastic deformation behavior of AZ31 magnesium alloy is worthy of understanding. In this work, the deformation behavior and mechanical response of an Mg AZ31 plate with {10–12} twins under biaxial tension is systematically investigated for the first time.

## 2. Experiments and Methods

### 2.1. Material and Mechanical Tests

A hot-rolled Mg AZ31 plate with a thickness of 60 mm was cut into blocks of 40 mm (RD) × 40 mm (TD) × 40 mm (ND, normal direction), and anneal-treated at 400 °C for 2 h to obtain a fully recrystallized structure. The chemical composition of the alloy is shown in Table 1.

**Figure 1:**
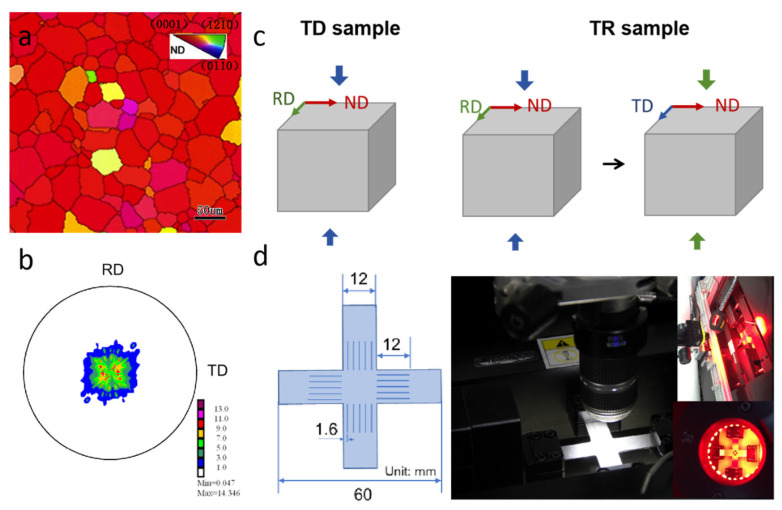
(**a**) ND inverse pole figure map and (**b**) pole figure of $\left(0001\right)\;\mathrm{and}\;\left(10\bar{1}0\right)$ for initial sample. (**c**) Schematic diagram of compression along TD (TD sample) and multi-directional compression along TD and RD (TR sample). (**d**) Cruciform specimen and IPBF-8000 (CARE Measurement & Control Co., Ltd., Tianjing, China) biaxial tension testing system.

The ND inverse pole figure map and pole figure of $\left(0001\right)\;and\;\left(10\bar{1}0\right)$ for the initial sample is shown in Figure 1a,b. The annealed sample has equiaxed grains with an average grain size of 40 μm. A texture was observed, with the *c*-axes of grains largely parallel to ND, and a random distribution of prismatic planes. The as-annealed sample was subjected to compression along the TD (TD sample) or cross compression along the TD and RD (TR sample), as schematically illustrated in Figure 1c. The samples for compression were subjected to a low strain (3.5%) along the TD, and a high strain (6.5%) along the RD was adopted. Some of the TD samples and TR samples were aged at 180 °C for about 0.5 h, named as the TDH sample and the TRH sample. According to a previous study [14], the as-used annealing can lead to solute atom segregation at the twin boundary and hardly affects the fraction of twins. All treatments for the TD sample, TR sample, TDH sample, and TRH sample are shown in Table 2. Then, the cruciform specimens were fabricated by laser cutting from the four samples with the x-axes parallel to TD (TD and TDH sample) or RD (TR and TRH sample), and the y-axes parallel to ND. Here, x and y are the loading directions during biaxial tension. The cruciform specimens have a gauge section of 12 mm × 12 mm and each arm has five slits with an interval of 2.2 mm, shown in Figure 1d. Generally, the slits in each arm were machined to exclude the geometric constraint on deformation. The rationality of cruciform specimen geometry has been researched by finite-element analysis (FEA) and digital image correlation (DIC) in our previous research [15].

Then, biaxial tensile tests at room temperature were carried out using an IPBF-8000 biaxial tension testing system, as shown in Figure 1d. The specimens were subjected to proportional loading along the x and y axes at room temperature with nominal stress ratios $\sigma_{xx}:\sigma_{yy}$ = 2:1, 1:1, and 1:2. For comparison, the uniaxial tension along the x axis and y axis at room temperature was also measured; the specimens for measuring uniaxial tension were dog-bone shaped with nominal gauge dimensions of 25 mm (length) × 5 mm (width) × 2 mm (thickness). The tension displacements were measured using an optical extension meter. Each mechanical test was repeated more than three times.

### 2.2. Characterization of Microstructure and Texture

In order to quantify the textures, the initial and deformed samples were examined by an electron back-scattered diffraction (EBSD) technique. The specimens for the EBSD recording were cut from the center of the gauge area and ground using SiC papers followed by electrochemical polishing in an AC2 electrolyte at 20 V for 90 s. All EBSD data were analyzed using Channel 5 software.

## 3. Results

### 3.1. Mechanical Behavior

**Figure 2:**
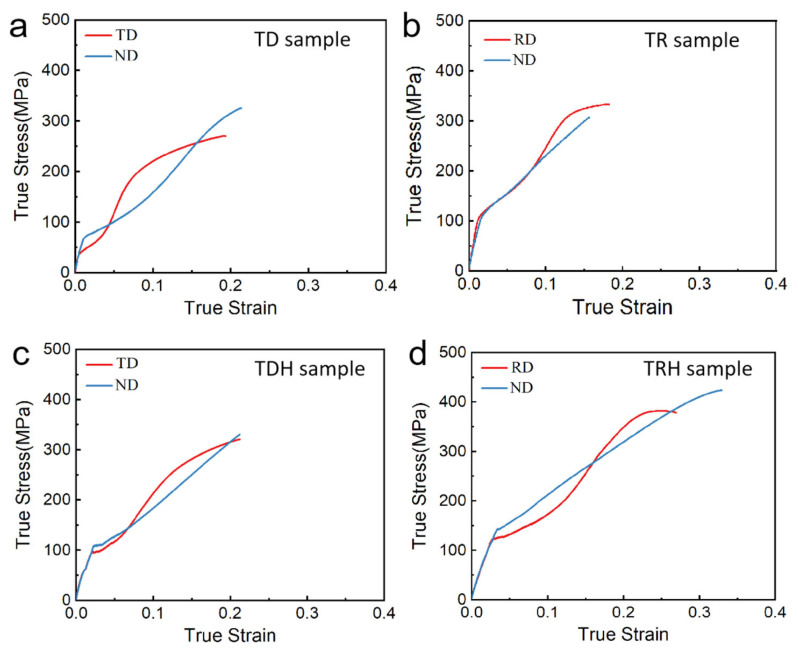
True stress–strain curves of the TD sample (**a**), TR sample (**b**), TDH sample (**c**) and TRH sample (**d**) under uniaxial tension.

The true strain–stress curves of samples under the uniaxial tension to fracture were shown in Figure 2. The mechanical properties of the TD sample, TR sample, TDH sample, and TRH sample in different directions were shown in Table 3. The yield strengths of TD samples along the TD and ND were 38.2 MPa and 70.6 MPa, respectively, showing an obvious yield anisotropy. The yield values of TR samples along the ND and RD were 108.5 MPa and 111.8 MPa, respectively, and the yield anisotropy decreased significantly. Compared with the TD sample, the yield strength of the TDH sample in both directions increased significantly after heat treatment. Compared with the TR sample, the yield strength of the TRH sample increased slightly in both directions after heat treatment.

**Figure 3:**
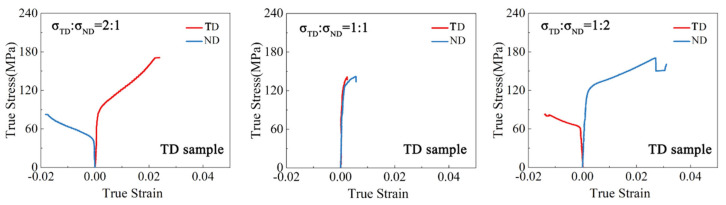
True stress–strain curves of the TD sample under biaxial tension.

**Figure 4:**
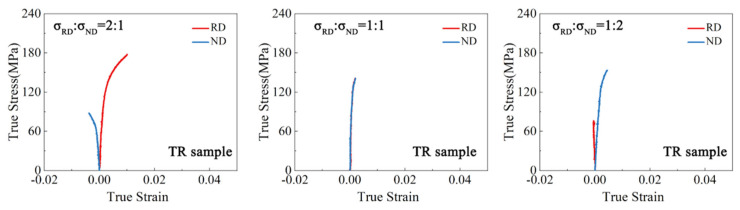
True stress–strain curves of the TR sample under biaxial tension.

**Figure 5:**
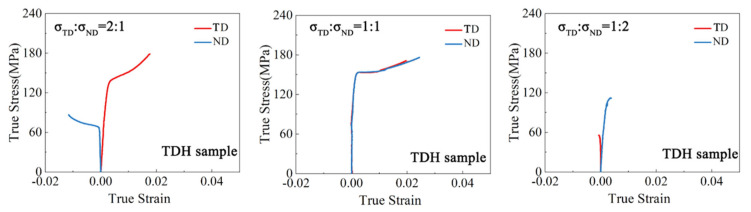
True stress–strain curves of the TDH sample under biaxial tension.

**Figure 6:**
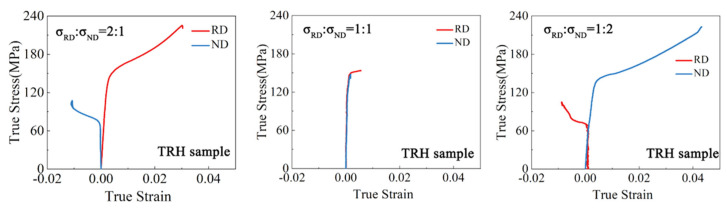
True stress–strain curves of the TRH sample under biaxial tension.

The true strain–stress curves of the samples under biaxial tension are depicted in Figure 3, Figure 4, Figure 5 and Figure 6. For both the TD and TR sample, under the stress ratio 1:1, the stress–strain curves in the two loading directions were similar and the plastic strain was very small. Under the stress ratio 2:1 and 1:2, a significant biaxial hardening was observed. Specifically, the yield values in the direction of larger stress values were higher than those under uniaxial loading conditions. In addition, the plasticity of the TR sample was significantly lower than that of the TD sample. For the TDH sample, the true stress–strain curves under biaxial loading were similar to those of the TD sample, but the yield strength was significantly increased. For the TRH sample, it can be seen that the strength and plasticity were significantly improved by annealing treatment, compared to the TR sample, noting that the strengthening effect of the RD yield stress for $\sigma_{\mathrm{RD}}:\sigma_{\mathrm{ND}}$ = 2:1 was higher than that of the ND yield stress under $\sigma_{\mathrm{RD}}:\sigma_{\mathrm{ND}}$ = 1:2.

### 3.2. Evolution of Microstructure

#### 3.2.1. Initial Microstructure

**Figure 7:**
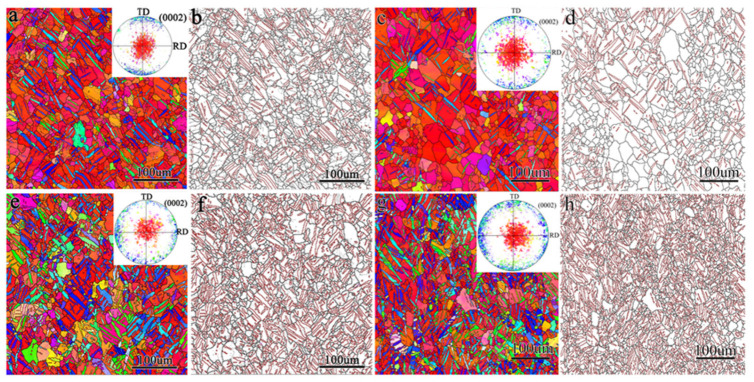
Inverse pole figure and grain boundary of the TD sample (**a**,**b**), TR sample (**c**,**d**), TDH sample (**e**,**f**), and TRH sample (**g**,**h**).

The microstructure of the TD sample, TR sample, TDH sample, and TRH sample is shown in Figure 7, and many twins are observed. For the TD sample, the twin morphology was relatively simple and mostly parallel to each other. The texture component changed from the original typical basal texture to two texture components with one component of (0002) close to the ND and the other of (0002) close to the TD. The twin volume fraction was about 20.1% (The volume fraction of the twins was measured as the area fraction of the twins in the EBSD map). For the TR sample, both the primary and secondary twins were detected, and the morphology of the primary twin was intersected. The twin volume fraction was about 42.5%. The texture was composed of three components with (0002) close to the ND, (0002) close to the TD and (0002) close to the RD, respectively. For the TDH and TRH samples, annealing treatment did not change the structure and texture.

#### 3.2.2. Microstructure of the Deformed Sample

**Figure 8:**
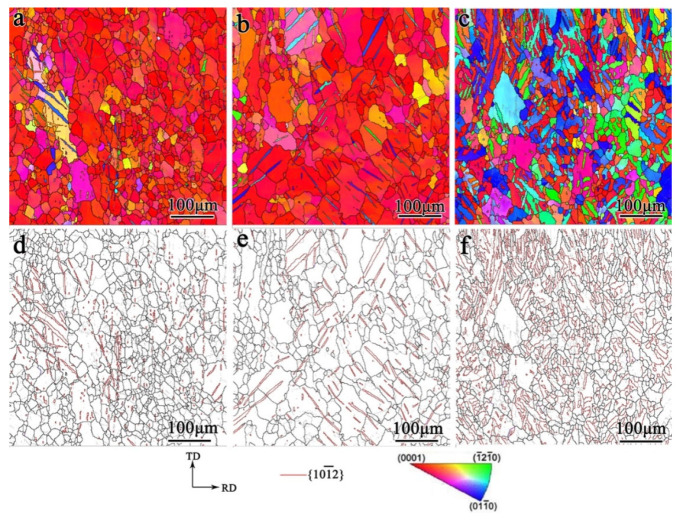
Inverse pole figure and grain boundary figure for TD sample under biaxial tension: (**a**,**d**) $\sigma_{\mathrm{TD}}:\sigma_{\mathrm{ND}}$ = 2:1; (**b**,**e**) $\sigma_{\mathrm{TD}}:\sigma_{\mathrm{ND}}$ = 1:1; (**c**,**f**) $\sigma_{\mathrm{TD}}:\sigma_{\mathrm{ND}}$ = 1:2.

**Figure 9:**
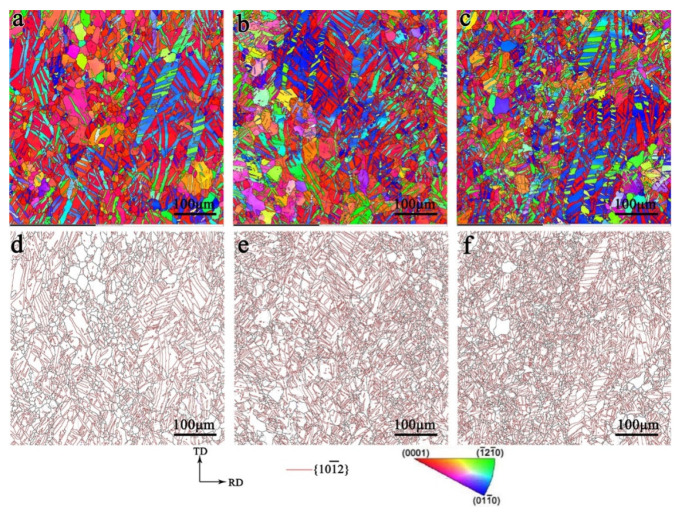
Inverse pole figure and grain boundary figure for TR sample under biaxial tensioned: (**a**,**d**) $\sigma_{\mathrm{RD}}:\sigma_{\mathrm{ND}}$ = 2:1; (**b**,**e**)$\sigma_{\mathrm{RD}}:\sigma_{\mathrm{ND}}$ = 1:1; (**c**,**f**) $\sigma_{\mathrm{RD}}:\sigma_{\mathrm{ND}}$ = 1:2.

**Figure 10:**
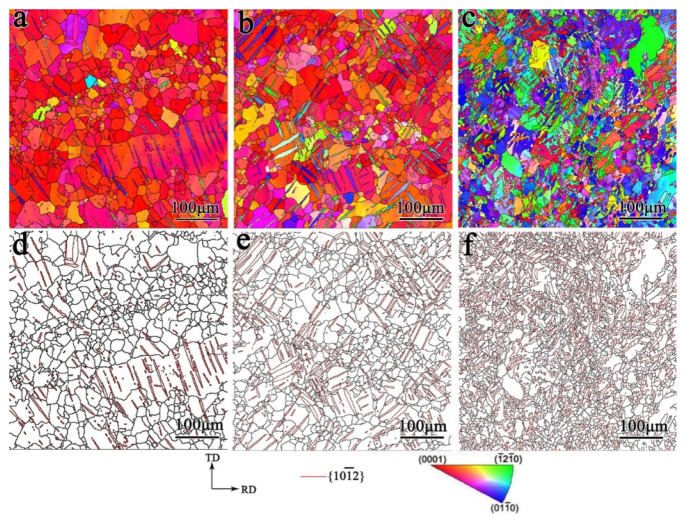
Inverse pole figure and grain boundary figure for TDH sample under biaxial tension: (**a**,**d**) $\sigma_{\mathrm{TD}}:\sigma_{\mathrm{ND}}$ = 2:1; (**b**,**e**) $\sigma_{\mathrm{TD}}:\sigma_{\mathrm{ND}}$ = 1:1; (**c**,**f**) $\sigma_{\mathrm{TD}}:\sigma_{\mathrm{ND}}$ = 1:2.

**Figure 11:**
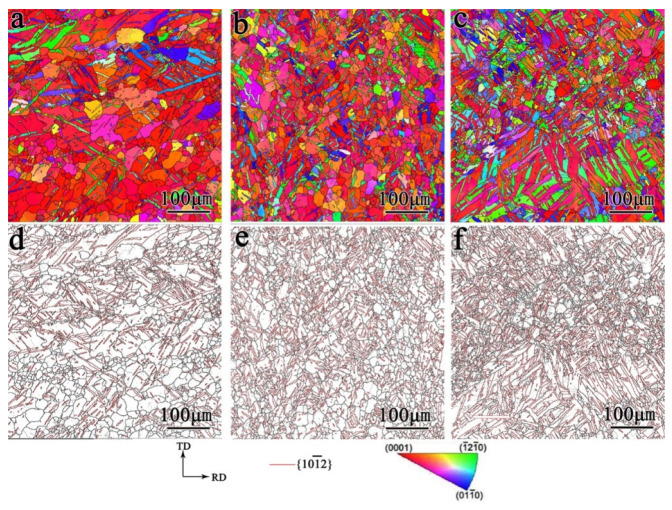
Inverse pole figure and grain boundary figure for TRH sample under biaxial tension: (**a**,**d**) $\sigma_{\mathrm{RD}}:\sigma_{\mathrm{ND}}$ = 2:1; (**b**,**e**) $\sigma_{\mathrm{RD}}:\sigma_{\mathrm{ND}}$ = 1:1; (**c**,**f**) $\sigma_{\mathrm{RD}}:\sigma_{\mathrm{ND}}$ = 1:2.

The microstructure of the samples under biaxial tensile deformation was examined and the results are shown in Figure 8, Figure 9, Figure 10 and Figure 11. As shown in Figure 8, under the stress ratio $\sigma_{\mathrm{TD}}:\sigma_{\mathrm{ND}}$ = 2:1, the twin volume fraction of the TD sample was significantly lower than that of the undeformed sample. Under the stress ratio $\sigma_{\mathrm{TD}}:\sigma_{\mathrm{ND}}$ = 1:1, a small number of twins were present, while the twins were much more numerous than for the stress ratio $\sigma_{\mathrm{TD}}:\sigma_{\mathrm{ND}}$ = 2:1. Hence detwinning occurred and the degree of detwinning increased with the increase in TD force. When $\sigma_{\mathrm{TD}}:\sigma_{\mathrm{ND}}$ = 1:2, the twin volume fraction increased significantly, up to 51.6%, which indicated that the twinning was active.

As shown in Figure 9, similar to the TD sample, the twin volume fraction of the TR sample decreased under the stress ratio $\sigma_{\mathrm{RD}}:\sigma_{\mathrm{ND}}$ = 2:1 and 1:1. Under the stress ratio $\sigma_{\mathrm{RD}}:\sigma_{\mathrm{ND}}$ = 1:2, the twin volume fraction increased significantly. However, the increased fraction of twins in the TR sample was smaller than that of the TD sample.

Figure 10 shows that the microstructure of the TDH sample after biaxial tension shows no obvious change compared with that of TD sample. Figure 11 shows that, compared to the TR sample structure, only a small number of secondary twin structures was observed in the TRH samples after biaxial tension.

### 3.3. Texture Evolution

**Figure 12:**
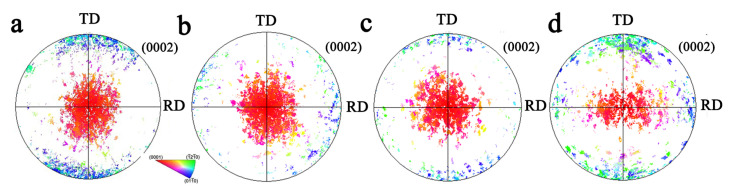
Pole figure for undeformed TD sample and biaxial-tensioned samples: (**a**) undeformed sample; (**b**) $\sigma_{\mathrm{TD}}:\sigma_{\mathrm{ND}}$ = 2:1; (**c**) $\sigma_{\mathrm{TD}}:\sigma_{\mathrm{ND}}$ = 1:1; (**d**) $\sigma_{\mathrm{TD}}:\sigma_{\mathrm{ND}}$ = 1:2.

**Figure 13:**
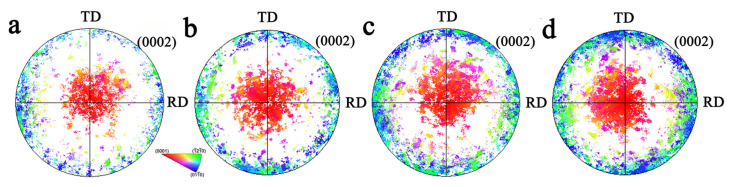
Pole figure for undeformed TR sample and biaxial-tensioned samples: (**a**) undeformed sample; (**b**) $\sigma_{\mathrm{RD}}:\sigma_{\mathrm{ND}}$ = 2:1; (**c**) $\sigma_{\mathrm{RD}}:\sigma_{\mathrm{ND}}$ = 1:1; (**d**) $\sigma_{\mathrm{RD}}:\sigma_{\mathrm{ND}}$ = 1:2.

Polar figures of TD samples and biaxial tensile samples under different stress ratios are shown in Figure 12. For $\sigma_{\mathrm{TD}}:\sigma_{\mathrm{ND}}$ = 2:1, there was almost no component with (0002) close to the TD. When $\sigma_{\mathrm{TD}}:\sigma_{\mathrm{ND}}$ = 1:1, the TD component was higher than that of $\sigma_{\mathrm{TD}}:\sigma_{\mathrm{ND}}$ = 2:1. When $\sigma_{\mathrm{TD}}:\sigma_{\mathrm{ND}}$ = 1:2, the TD texture component began to increase. The pole figure of the TRH sample is shown in Figure 13. It can be observed that, with the increase in ND force, the texture was more and more evenly distributed in the TD and RD directions.

**Figure 14:**
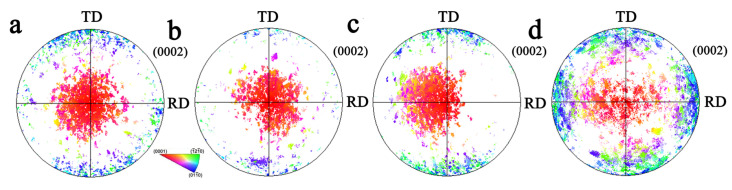
Pole figure for undeformed TDH sample and biaxial-tensioned samples: (**a**) undeformed sample; (**b**) $\sigma_{\mathrm{TD}}:\sigma_{\mathrm{ND}}$ = 2:1; (**c**) $\sigma_{\mathrm{TD}}:\sigma_{\mathrm{ND}}$ = 1:1; (**d**) $\sigma_{\mathrm{TD}}:\sigma_{\mathrm{ND}}$ = 1:2.

**Figure 15:**
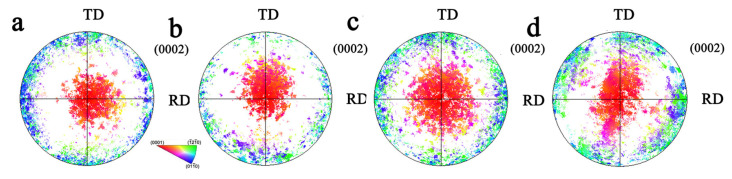
Pole figure for undeformed TRH sample and biaxial-tensioned samples: (**a**) undeformed sample; (**b**) $\sigma_{\mathrm{RD}}:\sigma_{\mathrm{ND}}$ = 2:1; (**c**) $\sigma_{\mathrm{RD}}:\sigma_{\mathrm{ND}}$ = 1:1; (**d**) $\sigma_{\mathrm{RD}}:\sigma_{\mathrm{ND}}$ = 1:2.

The polar figure of the TDH sample under different stress ratios is shown in Figure 14. Compared to the TD sample, the TD component of the deformed sample under biaxial stress ratio $\sigma_{\mathrm{TD}}:\sigma_{\mathrm{ND}}$ = 2:1 and 1:1 decreased significantly. The TD component reduction under $\sigma_{\mathrm{TD}}:\sigma_{\mathrm{ND}}$ = 2:1 is more effective than that of $\sigma_{\mathrm{TD}}:\sigma_{\mathrm{ND}}$ = 1:1. When $\sigma_{\mathrm{TD}}:\sigma_{\mathrm{ND}}$ = 1:2, compared to the TD sample, the RD and TD components increased. At the same time, with the decrease in the stress ratio, the ND component gradually decreased. The pole figures of the TRH sample and biaxial tensioned sample are shown in Figure 15. Compared to the undeformed sample, when the biaxial loading ratio $\sigma_{\mathrm{RD}}:\sigma_{\mathrm{ND}}$ = 2:1, the basal orientation near TD and RD decreased by different degrees. When $\sigma_{\mathrm{RD}}:\sigma_{\mathrm{ND}}$ = 1:1, the basal orientation near TD and RD increased slightly. Under the stress ratio of $\sigma_{\mathrm{RD}}:\sigma_{\mathrm{ND}}$ = 1:2, the basal orientation near ND changes greatly, with a greater degree close to TD.

## 4. Discussion

### 4.1. Competition Behavior between Twinning and Detwinning

**Figure 16:**
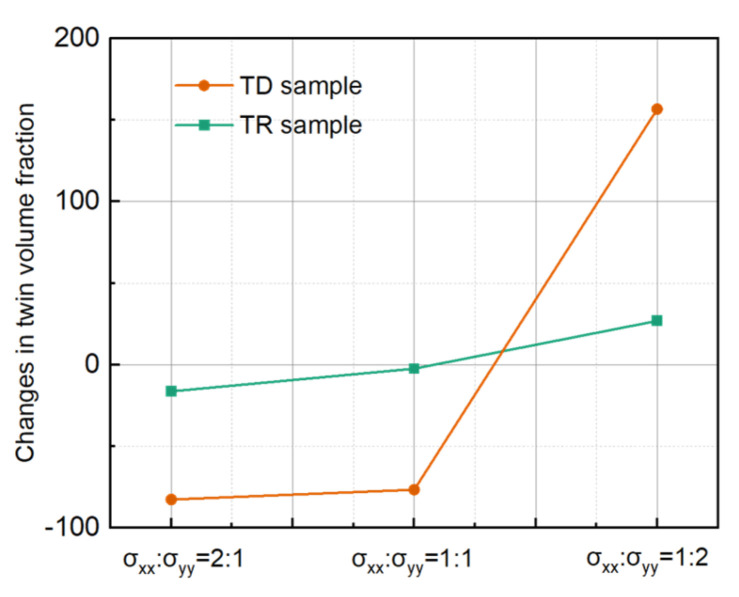
Changes in the twin volume fraction of TD and TR deformed samples compared with undeformed samples under different stress ratios.

Based on the microstructure evolution, it can be seen that the twin types of TD (primary twins) and TR (primary and secondary twins) samples are completely different. The evolution of the microstructure and the response of the mechanical behavior under biaxial tension is entirely different. In this section, the competitive behavior of twinning and detwinning under biaxial stress loading and the influence of twin types on the competitive behavior are studied. The competition between twinning and detwinning can be reflected by the twin volume fraction. Hence, the changes in the twin volume fraction of deformed TD and TR compared with undeformed samples under different stress ratios were analyzed, and the results are shown in Figure 16. In Figure 16, the negative sign indicates that the twin volume fraction decreases, while the positive sign indicates that the twin volume fraction increases. It can be seen from the figure that the variation trend in the twin volume fraction of the TD and TR sample after biaxial loading deformation first decreases and then increases with the increase in the ND direction force.

**Figure 17:**
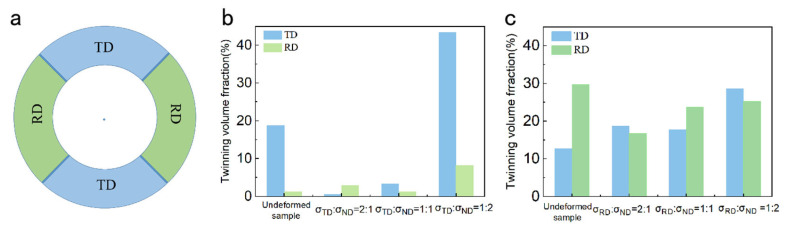
(**a**) Definition figure of TD and RD orientation and distribution of TD and RD orientation for TD (**b**) and TR (**c**) sample.

For the initial sample with the basal texture, the dominant deformation mechanism in TD compression is {10–12} twinning [16]. Therefore, for the TD sample prepared by compression along the TD, the ND texture components are mainly composed of the original grain and the residual parent. The TD texture components are mainly composed of {10–12} twins. Under the condition of biaxial loading, the stress along the TD direction mainly leads to detwinning or prismatic slip. The stress in the direction of ND has two functions. Firstly, it acts to promote the growth of twins, increasing the twin volume fraction, or causes nucleation of new twins. Therefore, under the biaxial tension along the TD and ND, the competition between twinning and detwinning occurs in the grain. It is well known that the stress required for detwinning is very small [17]. When the TD stress ratio is relatively large, detwinning will be the dominated mechanism. With the decrease in the stress ratio of TD, the residual twins grow further and the un-twinned grains will continue twinning. Therefore, the twin volume fraction first decreases and then increases again. The evolution of the twinning behavior can also be reflected by texture evolution. In order to further verify this conclusion, the method shown in Figure 17a was used in this paper to give statistical results for the proportions of texture components. Figure 17b shows that, under the stress ratio $\sigma_{\mathrm{TD}}:\sigma_{\mathrm{ND}}$ = 2:1 and 1:1, the proportion of TD texture components decreases. This further confirms the significant role of the detwinning. Under the stress ratio $\sigma_{\mathrm{TD}}:\sigma_{\mathrm{ND}}$ = 1:2, the proportion of TD texture components increases, which is due to the growth of the primary {10–12} twin. It is worth mentioning that, under all stress ratio conditions, RD texture components show a slight increase. Under biaxial tension along the TD and ND, the {10–12} twins induced by ND stress can turn to both the TD or RD. The larger force in the direction of ND contributes to the formation of twins, and thus the orientation of RD increases significantly.

During the preparation of the TR sample, the dominant deformation mechanism under TD compression is {10–12} twinning and the recompression along the RD is {10–12} twinning in the residual matrix and secondary twinning in the {10–12} primary twins. Therefore, the ND texture is composed of parent grain, the TD texture is composed of primary twin, and the RD texture is composed of primary twins and secondary twins. Under the biaxial tension along the RD and ND, the effect of the stress in the ND direction induces the twinning in the parent grain or causes the growth of the primary twins. The main role of RD stress leads to the detwinning of both the primary and secondary twins. When the RD direction stress is large, the detwinning is more active. With the decrease in stress ratio along the RD, the twin growth is dominant, so the twin volume fraction increases. The stress to activate secondary twinning is much larger due to the fine twins, therefore the detwinning is mainly observed in the primary twin. The proportions of texture components of the TR sample under different biaxial stress ratios are shown in Figure 17c. It can be seen that the proportion of the TD texture component increases and that of the RD component decreases. The decrease in the RD component can be ascribed to detwinning. There are two main reasons for the increase in the TD texture component. Firstly, the stress along the ND makes the initial twins oriented towards TD grow further. Secondly, the stress along the RD leads to secondary twinning and detwinning.

### 4.2. Effect of the Solute Atom on Twinning and Detwinning Behavior

**Figure 18:**
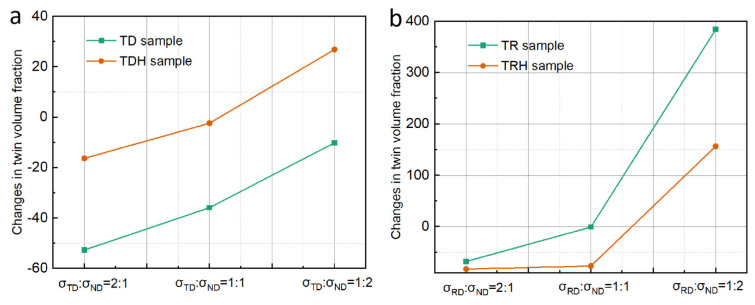
Change in twin volume fraction for (**a**) TD and TDH samples and (**b**) TR and TRH sample.

In this section, the influence of the solute atom on the competition between twinning and detwinning is discussed. The changes in the twin volume fraction of TD and TDH samples compared with undeformed samples under different loading ratios were analyzed, and the results obtained are shown in Figure 18a. The figure shows that, under the loading ratio of $\sigma_{\mathrm{TD}}:\sigma_{\mathrm{ND}}$ = 2:1 and 1:1, the decrease in the twin volume fraction is lower than that of the TD samples, indicating that annealing treatment can effectively inhibit the occurrence of detwinning to a certain extent. Previous studies have shown that proper heat treatment of the pre-deformed material can lead to the segregation of the solute atom at twin boundaries, which will pin the migration of the twin boundary, suppressing the detwinning of the {10–12} twin [18]. When $\sigma_{\mathrm{TD}}:\sigma_{\mathrm{ND}}$ = 1:2, the twin volume fraction of the TDH sample increases by about 384%, which is much higher than that of the TD sample (156.7%). This may be because the pinning effect of the solute atom is a much greater impact on detwinning and the growth of twins.

**Figure 19:**
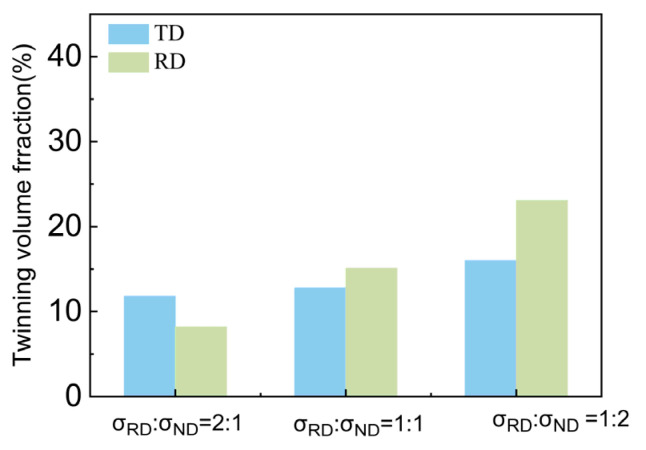
Distribution of TD and RD orientation for TRH sample.

Interestingly, the effect of annealing treatment on the TD and TDH samples is completely opposite, compared to that for the TR and TRH samples. The changes in twin volume fraction of TR and TRH samples under different loading ratios are shown in Figure 18b, where the negative values represent the decreasing amplitude and the positive values reflect the increasing amplitude. It can be observed that, under the stress ratio $\sigma_{\mathrm{RD}}:\sigma_{\mathrm{ND}}$ = 2:1 and 1:1, the decrease in the twin volume fraction of the TRH sample is higher than that of the TR sample, which is opposite when compared to the change in the TD and TDH sample. When $\sigma_{\mathrm{RD}}:\sigma_{\mathrm{ND}}$ = 1:2, the twin volume fraction of the TRH sample still decreases by about 10.11%, but the TR sample increases by about 26.89%. This may indicate that the twinning and detwinning behaviors are very complex in TRH samples containing multiple twin modes [19]. The evolution of the twinning behavior can be reflected by the texture evolution. In order to elucidate the cause of this extraordinary twinning and detwinning behavior, the proportions of texture components under different stress ratios of biaxial tensile were analyzed statistically. As shown in Figure 19, under the stress ratio $\sigma_{\mathrm{TD}}:\sigma_{\mathrm{ND}}$ = 2:1 and 1:1, the proportion of TD and RD texture components was lower than that of the TR sample (Figure 17c), which implies an increase in ND texture components. The ND texture component is closely related to the growth of primary twins towards TD and RD. Hence, the lower increase in the twin volume fraction of the TRH sample than that of the TR sample is closely related to the pinning effect of solute atoms on primary twins.

Under the stress ratio $\sigma_{\mathrm{TD}}:\sigma_{\mathrm{ND}}$ = 1:2, the proportion of TD texture components for the TRH sample was significantly lower than that of the TR sample. Therefore, the difference between the twin volume fraction of TRH and TR samples is closely related to the evolution of the TD texture component. The TD texture is closely related to the growth of primary twins towards TD and detwinning of the secondary twins towards RD. Due to the segregation of the solute atom, the growth of the primary twins and the detwinning of the secondary twins are restricted. Hence, the lower increase in the twin volume fraction of the TRH sample than that of the TR sample is closely related to the pinning effect of solute atoms on primary and secondary twin boundaries.

## 5. Conclusions

A comparative study of the biaxial deformation behavior of an Mg AZ31 plate containing four types of twin structure, {10–12} twins, {10–12} twins and secondary twins, {10–12} twins with solute atoms, and {10–12} twins and secondary twins with solute atoms, respectively, was systematically carried out for the first time. The main conclusions are as follows:

1. Significant hardening was observed under biaxial tension. The yield values in the direction of larger stress values were higher than those under uniaxial loading conditions. The solute atom segregation at twin boundaries generates a more obvious strengthening effect, noting that, for the TRH sample, the strength effect of the RD yield stress $\sigma_{\mathrm{RD}}:\sigma_{\mathrm{ND}}$ = 2:1 was higher than that of the ND yield stress under stress ratio $\sigma_{\mathrm{RD}}:\sigma_{\mathrm{ND}}$ = 1:2.
2. There is a complex competition between twinning and detwinning under biaxal tension along the ND and TD of the pre-twinned samples with the variation in the stress ratio along the TD and RD. The variation in the twin volume fractions for all samples under biaxial tension firstly decreases and then increases, with a higher stress ratio along the ND. As for the TDH sample, the changes in the twin volume fraction were lower than that of the TD sample. However, the amplitude of variation in the twin volume fraction of the TRH sample is higher than that of the TR sample. This is because the relative activity of detwinning decreases and that of twinning increases, as ND stress mainly leads to the growth of pre-twins and TD often promotes detwinning of primary twins. With a higher stress ratio along the ND, the activity of twinning deformation increases and that of detwinning decreases.

## Figures and Tables

**Table 1:** Chemical composition of AZ31 magnesium alloy (mass fraction, %).

Al	Zn	Mn	Cu	Ni	Si	Fe	Mg
2.47	0.93	0.3	0.005	0.002	0.06	0.002	Bal

**Table 2:** Treatments for the TD sample, TR sample, TDH sample, and TRH sample.

Sample Name	Treatments
TD sample	TD compression
TR sample	Cross compression along the TD and RD
TDH sample	TD compression and aging at 180 °C for about 0.5 h
TRH sample	Cross compression along the TD and RD +aging at 180 °C for about 0.5 h

**Table 3:** Mechanical properties of the TD sample, TR sample, TDH sample, and TRH sample in different directions.

	TD/RD		ND	
	YS/MPa	UTS/MPa	YS/MPa	UTS/MPa
TD sample	38.2	270.8	70.6	325.9
TR sample	108.5	334.0	111.8	307.2
TDH sample	96.3	321.1	109.2	332.8
TRH sample	123.8	382.0	136.0	424.8

## Data Availability

Data will be available on request to the corresponding author.
